# Last Hours of Abraham Lincoln

**Published:** 1865-05

**Authors:** C. S. Taft

**Affiliations:** Acting Ass’t Surgeon, U. S. A.


### LAST HOURS OF ABRAHAM LINCOLN.

By C. S. TAFT, Acting Ass't Surgeon, U. S. A.

The following brief report of the circumstances attending the assassination, last hours,and autopsy of the late President, will doubtless prove of much interest to the profession, and may be relied upon as correct in all particulars, the notes from which it is written having been submitted to comparison with others taken, and corrected by the highest authority.

While sitting in an orchestra chair at Ford's Theatre, on Friday evening, the 14th ult., about 10.30 P. M., I heard the sharp report of a pistol in the direction of the State box, and turning my head in that direction, saw a wild looking man jump from the box to the stage, heard him shout "*Sic semper tyrannis*," as he brandished a glittering knife in his right hand for an instant, and dart across the stage from sight.

A few moments of utterly indescribable confusion followed, amid which I heard a call for a surgeon. I leaped upon the stage, and was instantly lifted by a dozen pair of hands up to the President's box, a distance of twelve feet from the stage. When I entered the box, the President was lying upon the floor, surrounded by his wailing wife and several gentlemen who had entered from the dress circle. The respiration was inaudible and scarcely perceptible, and he was totally insensible. Ass't Surgeon Charles A. Leale, U. S. V., was in the box, and had caused the coat and vest to be cut off in searching for the wound. The wound in the head was soon found, but at that time there was no oozing from it.

Several gentlemen in the box were insisting upon having the President removed to his home, but Dr. Leale and myself protested against such a proceeding, and insisted upon his being carried to the nearest house. He was removed to a house opposite, and laid upon a bed in fifteen minutes from the time the shot was fired.

The wound was there examined, the finger being used as a probe, and the ball found to have passed beyond the reach of the finger into the brain. I put a teaspoonful of diluted brandy between the lips, which was swallowed with much difficulty; a half teaspoonful administered ten minutes afterward, was retained in the throat, without any effort being made to swallow it. The respiration now became labored; pulse 44, feeble, eyes entirely closed, the left pupil much contracted, the right widely dilated; total insensibility to light in both.

Surgeon-General Barnes and Robert K. Stone, M. D., the family physician, arrived and took charge of the case. At their suggestion, I administered a few drops of brandy to determine whether it could be swallowed, but as it was not, no further attempt was made. The left upper eyelid was swollen and dark from effused blood; this was observed a few minutes after his removal from the theatre. About thirty minutes after he was placed upon the bed, discoloration from effusion began in the internal canthus of the right eye, which became rapidly discolored and swollen, with great protrusion of the eye.

About 11.30, P. M., twitching of the facial muscles of the left side set in and continued some fifteen or twenty minutes, and the mouth was drawn slightly to the same side. Sinapisms over the entire anterior surface of the body were ordered, together with artificial heat to the extremities.

The wound began to ooze very soon after the patient was placed upon the bed, and continued to discharge blood and brain tissue until 5.30, A. M., when it ceased entirely; the head, in the meantime, being supported in such a position as to facilitate the discharge. The only surgical aid that could be rendered, consisted in maintaining the head in such a position as to facilitate the discharge of the wound, and in keeping the orifice free from coagulum.

Col. Crane, Surgeon, U. S. A., had charge of the head during a great part of the time, being relieved at intervals in this duty by myself. While the wound was discharging freely the respiration was easy; but the moment the discharge was arrested from any cause, it became at once labored.

It was also remarkable to observe the great difference in the character of the pulse whenever the orifice of the wound was freed from coagulum, and discharged freely; thus relieving, in a measure, the compression. This fact will account for the fluctuations in the pulse, as given in the subjoined notes.

About 2, A. M., an ordinary silver probe was introduced into the wound by the Surgeon-General. It met an obstruction about three inches from the external orifice, which was decided to be the plug of bone driven in from the skull and longed in the track of the ball. The probe passed by this obstruction, but was too short to follow the track the whole length. A long Nelaton probe was then procured and passed into the track of the wound for a distance of two inches beyond the plug of bone, when the ball was distinctly felt; passing beyond this, fragments of the orbital plate of the left orbit were felt. The ball made no mark upon the porcelain tip, and was afterwards found to be of exceedingly hard lead.

Some difference of opinion existed as to the exact position of the ball, but the autopsy confirmed the correctness of the diagnosis upon first exploration. No further attempt was made to explore the wound.

After the cessation of the bleeding from the wound, the respiration was stertorous up to the last breath, which was drawn at twenty-one minutes and fifty-five seconds past seven; the heart did not cease to beat until twenty-two minutes and ten seconds past seven. My hand was upon the heart, and my eye on the watch of the Surgeon General, who was standing by my side, with his finger on the carotid.

The decubitus during the whole time was dorsal, and the position on the bed diagonal; the length of the bedstead not admitting of any other position.

The respiration during the last thirty minutes was characterized by occasional intermissions; no respiration being made for nearly a minute, but by a convulsive effort air would gain admission to the lungs, when regular, though stertorous, respiration would go on for some seconds, to be followed by another period of perfect repose.

At these times the death-like stillness and suspense were thrilling. The Cabinet ministers, and others surrounding the death-bed, watching, with suspended breath, the last feeble inspiration, and as the unbroken quiet would seem to prove that life had fled, turn their eyes to their watches; then as the struggling life within would force another fluttering respiration, heave deep sighs of relief, and fix their eyes once more upon the face of their dying chief.

The wonderful vitality exhibited by the late President, was one of the most interesting and remarkable circumstances connected with the case. It was the opinion of the surgeons in charge, that most patients would have died in two hours from the reception of such an injury, yet Mr. Lincoln lived from 10.30, P. M., until 7.22, A. M.

The following observations of the pulse and respiration were noted down by Dr. A. F. A. King, at the bed-side, and are correct. The pulse was counted by Acting Ass't Surgeon Ford:

10.55—48. 11.06—45. 11.18—42, and weaker.
11.24—42, respirations 27 per minute, breathing quiet.
11.26— irregular, intermits occasionally.
11.30—45, respiration more frequent and vigorous.
11.32—45, stronger, resp. much more strong and stertorous.
11.39—48, respiration again silent and feeble.
11.40—45. 11.43—45, resp. stertorous.
11.47—45, resp. 24, stertorous. 11.56—48, weaker.
12.10—48, irregularly intermittent. 12.18—48, same.
12.27—54. 12.28—60. 12.29—66, intermittent.
12.38—66. 12.45—69, intermittent. 12.49—84, resp. 28.
12.56—66. 1.00—100. 1.15—92. 1.30—95.
2.10—60, resp. 34. 2.19—58. 2.32—54. 2.37—48.
2.54—48, much weaker, more thready; resp. feeble.
4.18—60, resp. 27, strong and stertorous.
5.40—64, thready, resp. 27.
6.10—60, hardly perceptible, (Barnes,) resp. 26, ster.
6.25— thready, not counted; resp. 25; insp. jerking.
6.40— insp. short and feeble; exp. prolonged and groaning; a deep, softly sonorous, cooing sound at the end of each exp., audible to bystanders.
6.45— resp. uneasy, choking and grunting; lower jaw relaxed; mouth open; a minute without a breath; face getting dark.
6.59— breathes again a little more at intervals; another long pause.
7.00— still breathing at long pauses.
7.20— died.

About 1, P. M., spasmodic contractions of the muscles came on, causing pronation of the forearms; the pectoral muscles seemed to be fixed, the breath was held during the spasm, and a sudden and forcible expiration immediately succeeded it.

At about the same time both pupils became widely dilated, and remained so until death.

During the night Drs. Hall, May, Liebermann, and nearly all the leading men of the profession in the city, tendered their services.

AUTOPSY—FIVE HOURS AFTER DEATH.

Present, Surgeon-General Barnes, Col. Crane, Dr. Stone, Ass't Surg. Woodward, U. S. A., Ass't Surg. Curtis, U. S.A., Ass't Surg. Notson, U. S. A., and Acting Ass't Surg. Taft, U. S. A.

The calvaria was removed, the brain exposed, and sliced down to the track of the ball, which was plainly indicated by a line of coagulated blood, extending from the external wound in the occipital bone, obliquely across from left to right through the brain to the anterior lobe of the cerebrum, immediately behind the right orbit. The surface of the right hemisphere was covered with coagulated blood. After removing the brain from the cranium, the ball dropped from its lodgment in the anterior lobe. A small piece of the ball, evidently cut off in its passage through the occipital bone, was previously taken out of the track of the ball, about four inches from the external wound. The hole made through the occipital bone was as cleanly cut as if done with a punch.

The point of entrance was one inch to the left of the longitudinal sinus. The ball was flattened, convex on both sides, and evidently moulded by hand in a Derringer pistol mould, as indicated by the ridged surface left by the nippers in clipping off the neck.

The orbital plates of both orbits were the seats of comminuted fracture, the fragments being forced inward, and the dura mater covering them remaining uninjured. The double fracture was decided to have been caused by *contre coup*. The plug of bone driven in from the occipital bone, was found in the track of the ball, about three inches from the external wound, proving the correctness of the opinion advanced by the Surgeon-General and Dr. Stone as to its nature, at the exploration of the wound before death.

The ball and fragments, together with the fragments of the orbital plates and plug from the occipital bone, were placed in the possession of Dr. Stone, the family physician, who marked and delivered them, pursuant to instructions, to the Secretary of State, who sealed them up with his private seal. The Nelaton probe used was also marked by me, and sealed up in like manner.—*Med. and Surg. Rep.*

